# Management of intraoperatively identified small bile duct stones in patients undergoing cholecystectomy

**DOI:** 10.1007/s00423-024-03260-9

**Published:** 2024-02-22

**Authors:** David Bunting, Abidemi Adesuyi, John Findlay, Maciej Pawlak, David Sanders

**Affiliations:** 1 Department of Upper GI and Abdominal Wall Surgery, North Devon District Hospital, Barnstaple, Devon EX31 4JB UK; 2 University of Exeter Medical School, Exeter, EX1 2HZ UK

**Keywords:** Common bile duct calculi, Ultrasound, Cholecystectomy

## Abstract

**Introduction:**

The management of CBDS (common bile duct stones) in patients with co-existing gallbladder stones has been debated. Guidelines recommend patients with CBDS identified on imaging should be offered duct clearance; however, this is based on low-quality evidence. This study aimed to investigate the natural history of small CBDS identified using IOUS (intraoperative ultrasound) in patients undergoing cholecystectomy. This may provide evidence to support a short-term expectant management approach in such patients.

**Methods:**

Patients with CBDS diagnosed on IOUS during cholecystectomy were identified from a database of consecutive patients undergoing surgery. Patients with CBDS identified were divided into small stone (SS, ≤5 mm) and large stone (LS, >5 mm) groups. Intraoperative CBDS management, postoperative investigations, postoperative bile duct clearance, re-admissions, complications, length of stay (LOS) and follow-up are described.

**Results:**

Fifty-nine of 427 patients had CBDS identified on IOUS. In the SS group (*n*=51), 46 patients underwent short-term expectant management rather than immediate/planned bile duct clearance. Following short-term expectant management, 41/46 patients (89.1%) did not require postoperative endoscopic retrograde cholangiopancreatography and at >3 year follow-up, none has since presented with residual CBDS. Median LOS was 0 days in the short-term expectant management group and 2 days in the immediate/planned bile duct clearance group, *P=*0.039.

**Conclusions:**

This study reports the natural history of small CBDS identified on IOUS in patients undergoing cholecystectomy. Such patients were safely treated with short-term expectant management associated with a reduced hospital LOS. This provides rationale for undertaking further research to establish this as a preferred management strategy.

## Introduction

### Common bile duct stones (CBDS)

Laparoscopic cholecystectomy (LC) has been embraced worldwide as the gold standard in the management of symptomatic gall stone disease since its introduction to the surgical armamentarium in the twentieth century. It represents the most commonly performed hepatobiliary procedure in the UK and the world at large. Data from the literature show that common bile duct stones (CBDS) occur in about 10 to 20% of patients with symptomatic gall stone disease 1, 2. In patients undergoing cholecystectomy without clinical suspicion of ductal stones preoperatively, the incidence of CBDS is typically less than 5% [1].

### Natural history and treatment

The management of common bile duct stones in patients with co-existing gallbladder stones has been debated. Current guidelines recommend patients with CBDS identified on imaging should be offered bile duct stone clearance [1–3]. This can be achieved either surgically at the time of laparoscopic cholecystectomy or with endoscopic retrograde cholangiopancreatography (ERCP) ^nice^. However, it is acknowledged that this guidance is based on low-quality evidence from symptomatic patients and expert opinion only [1]. Guidance also do not differentiate between patient with symptomatic and asymptomatic CBDS [1–3].

The natural history of CBDS is not well understood. Whilst they can lead to significant complications including pain, biliary obstruction, cholangitis, pancreatitis, biliary cirrhosis and liver abscesses, not all patients will suffer these and CBDS may pass spontaneously into the bowel without significant symptoms [4, 5]. Studies have shown that stones with diameters <5 mm are more likely to pass spontaneously [6]. Attempted bile duct clearance either by ERCP or operative bile duct exploration is associated with potential morbidity; therefore, doubt remains over whether all patients with CBD stones should have attempted duct clearance [7]. A short-term expectant approach in the management of clinically silent CBDS in patients selected for cholecystectomy has been suggested in a study of patients undergoing cholangiography intraoperatively and postoperatively [4]. Furthermore, with a proposed short-term expectant approach to the management of intraoperatively diagnosed CBDS, there is no consensus on what methods of follow-up are required whether that be clinical evaluation, monitoring of serum liver function tests (LFTs) or scheduled postoperative bile duct imaging.

### Diagnosis of CBDS

#### Preoperative imaging

Preoperative imaging such as ultrasound (US), magnetic resonance cholangiopancreatography (MRCP), abdominal computed tomography (CT) and endoscopic ultrasound scan (EUS) are performed based on the patient’s risk of CBDS as recommended by the British Society of Gastroenterologists (BSG) guidelines [1].

#### Intraoperative imaging

Intraoperative cholangiography (IOC) is used to identify CBDS and to clarify biliary tract anatomy in an effort to reduce the chance of bile duct injury. However, despite its use over several decades, there is a lack of evidence, in favour of, or against, its routine use for either indication [1, 2]. As a result, many surgeons prefer selective use of IOC; or they may consider alternative, less invasive biliary tract imaging such as preoperative MRCP; or they may offer no targeted bile duct imaging at all.

Intraoperative ultrasound (IOUS) is an alternative imaging modality for the biliary tract, with reported advantages over IOC. These include being quicker to perform, a lower failure rate, no use of ionising radiation, ease of repeating during a procedure, the ability to use prior to Calot’s triangle dissection, less risk of cystic duct avulsion and lower cost [8–11]. A basic knowledge of ultrasound, image interpretation and clinical correlation are required, which is associated with a learning curve. Reports demonstrate that compared with IOC, IOUS is at least as accurate in the detection of CBDS with a sensitivity and specificity of 80–92% and 99–100%, respectively, in the hands of trained users [8, 10]. This has resulted in an increase in the use of selective and routine intraoperative use of IOUS in some centres, although despite perceived advantages, it is still not widely available or used routinely in the majority of centres [9].

According to BSG and World Society of Emergency Surgeons (WSES) guidelines, imaging by IOUS or IOC is not mandatory for all patients undergoing cholecystectomy but is suggested for patients with intermediate/high probability of CBDS who have not had the diagnosis confirmed preoperatively by USS, MRCP or EUS. However, this is a weak recommendation based on low-quality evidence [1, 2].

A need for further research has been called for into the natural history of CBDS, particularly in asymptomatic patients in whom extraction is not performed [1]. Studies are required to determine whether bile duct clearance is beneficial for all patients with intraoperatively identified CBDS or whether certain patients, such as those with small stones, can be managed with a short-term expectant approach [4].

Due to the risks associated with IOC and potential for overtreatment of incidentally found CBDS, further research has been called for to clarify the harm/benefit of routine use of IOC [1]. If an evidence-based clinical management algorithm for managing intraoperatively identified CBDS stones included an expectant approach in selected patients, then routine use of IOUS could be safely employed without the risk of increased procedural morbidity/mortality or overtreatment.

Whilst studies have been performed to investigate the natural history of small CBDS identified on IOC in patients undergoing cholecystectomy using postoperative tube cholangiography [4], this has not been investigated in the context of more modern, less invasive techniques such as IOUS and postoperative MRCP.

### Aims

This study aimed to investigate the natural history of small CBDS identified using IOUS in patients undergoing cholecystectomy. This may provide evidence to support a short-term expectant management approach in such patients.

## Methods

A retrospective study was conducted at the Academic Department of Abdominal Wall and Upper Gastrointestinal Surgery at North Devon District Hospital, Royal Devon University Healthcare NHS Foundation Trust, UK, from a cohort of consecutive patients undergoing IOUS with cholecystectomy between December 2016 and April 2020 inclusive.

Institutional review board (IRB) approval and written consent were not required for this study.

This was a retrospective analysis of consecutive patients undergoing cholecystectomy for symptomatic cholelithiasis. IOUS was performed routinely. All patients were operated on by one of six consultant upper gastrointestinal surgeons. Patients in the cohort underwent preoperative CBDS imaging using MRCP on a selective basis according to clinical, biochemical and ultrasound scan findings. Patients with CBDS diagnosed preoperatively were either treated with preoperative ERCP or proceeded to cholecystectomy with IOUS ± bile duct exploration (BDE). CBDS identified on IOUS were treated with selective bile duct exploration, postoperative ERCP or short-term expectant management with or without postoperative bile duct imaging (MRCP) according to patient factors, IOUS finding and surgeons’ preference.

Data were recorded on patient demographics: IOUS-determined parameters (documented at the time of surgery: presence of CBD stones, CBDS stone number, largest CBDS diameter, CBD diameter); intraoperative details (bile duct exploration; placement of drains/t-tubes; total/sub-total cholecystectomy, laparoscopic/open); postoperative imaging; postoperative ERCP; and length of stay and follow-up details including re-admissions, mortality and further procedures.

The combined incidence of CBDS in this cohort of patients undergoing cholecystectomy for gallstone disease with IOUS was calculated by including patients treated preoperatively for CBDS, together with those having CBDS identified intraoperatively and those with CBDS diagnosed at any time after cholecystectomy for the duration of the follow up period. The rate of detection of CBDS diagnosed intraoperatively (intraoperative CBDS incidence) was calculated by dividing the number of patients with CBDS identified intraoperatively by the total cohort number after excluding patients undergoing preoperative ERCP. The rate of true incidental CBDS detection (those not suspected on the basis of preoperative imaging findings) was calculated by dividing the number of patients with CBDS identified intraoperatively by the total cohort number after excluding patients with CBDS diagnosed on preoperative MRCP or ERCP.

Patients with CBDS identified on IOUS were selected from the cohort and divided into two groups depending on largest CBDS diameter: small stone (SS) group, ≤5 mm, and large stone (LS) group, >5 mm.

Intraoperative CBDS management, postoperative investigation (serum LFTs, MRCP), postoperative bile duct clearance (ERCP), re-admissions, complications and follow-up were described.

Total hospital length of stay (LOS) was calculated in SS group patients by combining the LOS for index operation with the LOS relating to any separate admissions for postoperative ERCP. Patients were sub-divided into those undergoing short-term expectant management and those undergoing alternative management (int-operative BDE or post-op ERCP). Median total hospital LOS was calculated as for each group and LOSs in the two groups were compared using the two-tailed Mann-Whitney *U* test with a significance level of 95%.

## Results

A total of 427 patients underwent cholecystectomy with IOUS between December 2016 and April 2020 inclusive. Mean duration of follow-up was 3 years and 4 months. The combined incidence of CBDS was 20.4%, the intraoperative CBDS incidence was 12.5% and the rate of true incidental intraoperative CBDS detection was 11.0%; see Table 1. Sixty-seven patients had CBDS identified either preoperatively or on IOUS and 11 (16.4%) of these underwent postoperative CBD clearance.

**Table 1:** Patient demographics and operative data

Demographic data	All patients, *n*=427	CBDS, *n*=59	No CBDS, *n*=368
Age, mean (range) in years	55 (13–89)	56 (18–82)	55 (13–89)
Gender, female/male	314:113	33:26	281:87
CBDS combined incidence	20.4%	-	-
CBDS intraoperative incidence	12.5%	-	-
CBDS incidental detection rate	11.0%	-	-
CBD exploration, yes/no	9:421	9:47	0:55
Laparoscopic/open	426:1	58:1	368:0
Drain, yes/no	88:339	27:32	61:307
CBD dilatation, yes/no	108:319	37:22	71:297
Procedure, TC:STC	413:14	54:5	359:9
CBD size, mean, in mm	5.8	7.4	5.8
CBD size, median, in mm	5	7	5
CBD size, range, in mm	2–16	3–16	2–14

*CBDS* common bile duct stones, *CBD* common bile duct, *TC* total cholecystectomy, *STC* subtotal cholecystectomy

**Fig. 1:**
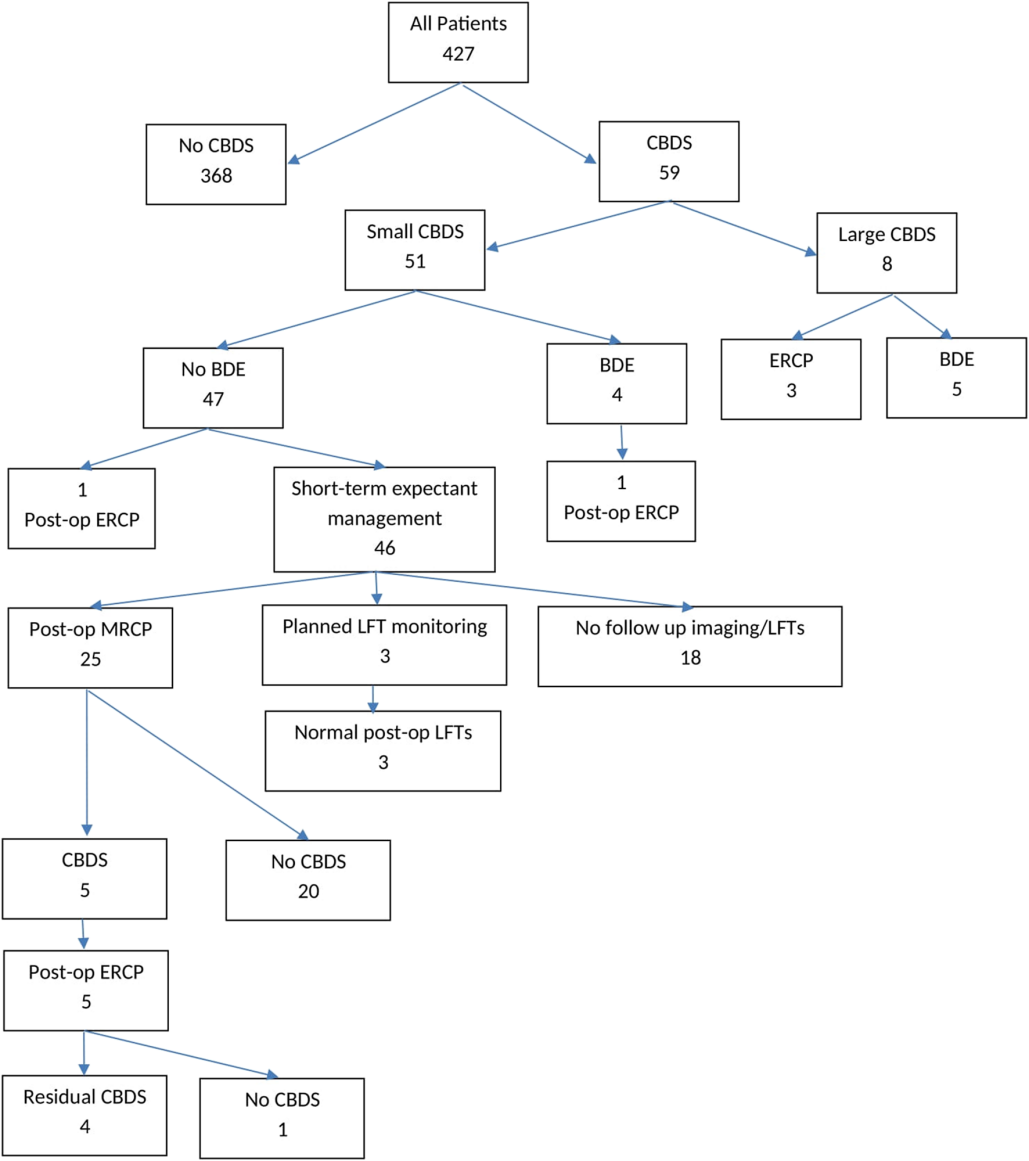
Study patients with intra-operatively identified CBDS. CBDS, common bile duct stones; BDE, bile duct exploration; ERCP, endoscopic retrograde cholangiopancreatography; MRCP, magnetic resonance cholangiopancreatography; LFT, liver function tests

Fifty-nine patients had CBDS identified on IOUS, 51 patients had CBDS ≤5 mm (SS group) and 8 patients had CBDS >5 mm (LS group); see Fig. 1.

### SS group

Within the SS group (Fig. 1), 4 patients underwent BDE and 47 did not. In the latter group, one patient underwent planned postoperative ERCP. Forty-six patients remained in the short-term expectant management group. Of these, 25 patients underwent planned postoperative MRCP (mean 5.8 weeks post op, range 0–12 weeks); 3 patients had planned LFT monitoring, and 18 patients were planned to have no follow up imaging or LFTs.

#### Bile duct exploration

Four patients underwent bile duct exploration and stone extraction at the time of surgery. One of these patients underwent postoperative ERCP for a suspected bile leak, with no persistent leak or residual CBDS found. No patients have re-presented with signs or symptoms of residual CBDS (mean follow-up duration 3 years and 2 months).

#### Planned post-op ERCP

One patient underwent planned postoperative ERCP with no stones seen on cholangiogram and a clear balloon trawl.

#### Short-term expectant management

Of the 25 patients undergoing postoperative MRCP, 20 patients had no evidence of residual CBDS, and 5 patients had CBD stones. All the latter patients underwent ERCP within 8 weeks of MRCP without complication. Four patients had extraction of confirmed CBDS, and one patient had no evidence of residual CBDS.

Of the three patients undergoing postoperative LFT monitoring, none had raised bilirubin levels postoperatively. At follow-up, none has re-presented with signs or symptoms of residual CBDS (mean follow-up duration, 4 years and 9 months).

Of the 18 patients with no follow-up planned, none has re-presented with signs or have symptoms of residual CBDS (mean follow up duration, 3 years and 1 month).

Following short-term expectant management as described above, 41/46 patients (89.1%, confidence interval, 76.4–96.3%) did not require postoperative endoscopic retrograde cholangiopancreatography (ERCP) and none has since presented with residual CBDS (mean follow up duration: 3 years and 5 months).

Total hospital LOSs for the short-term expectant management group was 0 days (range 0–31 days) compared with 2 days (range 0–10 days) in the alternative management group (BDE/ERCP), *P*=0.039 (Mann-Whitney *U* test).

### LS group

Within the LS group, five underwent BDE at the time of surgery and three did not. In the latter group, three patients underwent planned postoperative ERCP and stone extraction without complication.

At follow-up, no LS group patients have re-presented with signs or symptoms of residual CBDS (mean follow-up duration 3 years and 4 months).

## Discussion

IOUS is increasingly being used as a method of selectively or routinely delineating biliary anatomy and examining for bile duct stones during cholecystectomy due to its safety, ease use and other potential advantages over IOC [9–11]. The natural history of small CBDS identified using IOUS in patients undergoing cholecystectomy is poorly understood and, therefore, the optimal management of CBDS in the patients is not known. This study in a group of patients undergoing consecutive cholecystectomy with routine IOUS aimed to investigate the natural history of small CBDS (<5 mm) identified at the time of surgery. This threshold was chosen based on published evidence that stones with a diameter of less than 5 mm are more likely to pass spontaneously [6].

In our unit, many patients with small CBDS identified at the time of surgery through routine use of IOUS have been treated with short-term expectant management rather than undergoing intraoperative or postoperative bile duct clearance as first-line management of their CBDS. This approach has been previously published [4]. Short-term expectant management can include options such as no follow-up, clinical outpatient follow-up, scheduled monitoring of LFTs or planned biliary imaging (e.g. MRCP).

In our cohort of 427 patients undergoing cholecystectomy, the rate of intraoperative CBDS detection was 12.5%. This is in line with published rates in the literature of 5–15% [12]. The wide range of reported rates in the literature probably reflects different selection criteria for surgery, variation in the rates of acute biliary presentations and different strategies in both preoperative imaging and preoperative treatment of CBDS. When patients with CBDS identified and treated preoperatively are included, the overall rate of CBDS was 20.4%. This prevalence of CBDS in symptomatic gallstones is at the upper end of the range reported in the literature (10 to 20% [13]), which may reflect the fact that our unit runs a ‘hot-gallbladder’ service undertaking cholecystectomy with 2 weeks of acute presentation accounting for around one third of all cholecystectomies.

Of 427 patients, 46 had short-term expectant management of small CBDS identified on IOUS. It is routine in many institutions to proceed with operative (bile duct exploration) or postoperative (ERCP) clearance and current guidelines recommend offering this approach, although evidence for this is weak and largely based on patients with symptomatic CBDS. In our patients, only 4/46 (8.7%) patients in the short-term expectant management group were found to have persisting CBDS requiring treatment with ERCP as part of their planned follow-up. No patients had delayed presentation due to CBDS in the follow-up period (mean duration of 3 years and 5 months). Twenty-five patients had planned 6-week postoperative MRCP as part of their management plan and 21 did not. These decisions were made according to individual surgeon choice rather than selectively according to any patient or operative criteria. Interestingly, in the group not undergoing MRCP, no patients presented with symptoms due to CBDS in the follow-up period. In those with stones identified on MRCP and treated with ERCP, the natural history if they had not undergone duct clearance is not known.

A significant proportion (89.1%) of patients with short-term expectant management of small CBDS identified intraoperatively did not require postoperative endoscopic retrograde cholangiopancreatography (ERCP) and none has since presented with signs or symptoms of residual CBDS at long-term follow-up. Due to the unique methodology of our study, there are no published studies with which to make a direct comparison. The one published study on short-term expectant management of CBDS used IOC rather than IOUS and left bile duct catheters in place for delayed cholangiogram imaging at 48 h and 6 weeks [4]. They also included patients with CBDS of any size rather than limiting their protocol to small stones. 56.5% patients in this study did not undergo postoperative CBDS removal, significantly fewer than our study, which may be due to the inclusion of large CBDS [4]. Our data support a rationale for not proceeding with immediate/urgent bile duct clearance in patients with small CBDS identified at cholecystectomy by IOUS.

Total hospital LOS was shorter in patients undergoing short-term expectant management of small CBDS identified on IOUS compared with that in patients undergoing immediate/planned bile duct clearance. This is probably due to the fact that most patients undergoing intraoperative BDE require overnight stay following surgery. Reasons for this may include the placement of a drain intraoperatively, monitoring for the presence of a bile leak/sepsis, operative duration or surgeon’s preference for other reasons.

The authors recognise that small CBDS identified incidentally at the time of cholecystectomy could be treated with a trans-cystic bile duct approach to bile duct exploration, which is associated with somewhat fewer risks than choledochotomy. This is indeed a valid treatment option; however, the expertise is not universally available and the technique is not widely undertaken. Furthermore, it prolongs the operation, and it is not without any additional risk. If it was proven that small stones could be managed with expectant management, then many patients/surgeons may still wish to consider this as a treatment option.

Short-term expectant management of patients with small CBDS identified on IOUS could benefit both patients and the healthcare industry because it may be associated with reduced procedural-related morbidity, reduced hospital length of stay and reduced hospital costs.

### Limitations

This study is limited by not randomising patients to different treatment arms; therefore, it is not possible to state whether short-term expectant management reduces procedure-related patient morbidity, hospital LOS or hospital costs compared with immediate/planned bile duct clearance. This was a study with management determined by surgeons’ preferences rather than a prospective protocol so outcomes cannot be assumed to be related to management decisions. There were a relatively small number of patients in the short-term management group; larger numbers determined by a power calculation would be required for a study to make treatment recommendations. The natural history in patients with persisting CBDS identified on planned postoperative imaging is not known from this study as these patients underwent duct clearance. Likewise, a small number of patients with small CBDS detected on IOUS did have CBD clearance at some stage. The natural history in this specific group of patients is not known and this could be addressed in a prospective study. In patients with small CBDS undergoing expectant management without postoperative bile duct imaging, we only know they did not succumb to development of symptoms or require intervention and not whether there was radiologically confirmed spontaneous stone passage. There may be an argument to leave in situ small CBDS confirmed on postoperative MRCP in asymptomatic patients and a separate prospective study could be undertaken to address this.

One might consider a different approach to the management of intraoperatively identified small CBDS in patients with a prior presentation with abnormal LFTs/conservatively managed cholangitis/pancreatitis. In this cohort, as would be routine practice in many units, such patients have systematically undergone preoperative bile duct imaging and proceeded to ERCP prior to surgery or planned bile duct exploration; therefore, the incidence of intraoperatively identified small CBDS is low. Our cohort did include a number of such patients, all of whom were included in the analysis, providing some evidence that a prior presentation with deranged LFTs/cholangitis/pancreatitis does not necessarily preclude successful treatment with short-term expectant management.

We suggest further studies are required to investigate patients with CBDS identified on IOUS. These should include large-scale prospective cohort studies to examine the natural history of small CBDS identified on IOUS with planned short-term expectant management utilising postoperative biliary imaging (6-week MRCP). Randomised trials are also required to compare planned bile duct clearance (intraoperative or postoperative) with short-term expectant management utilising postoperative biliary imaging (6-week MRCP) in patients with small CBDS identified on IOUS. This study does not aim to investigate the natural history of large (>5 mm) CBDS or make any recommendation on their treatment and the authors would support management according to published guidelines, which generally recommend CBDS clearance.

Although in our small cohort, patients not undergoing postoperative imaging did not suffer any complications from residual CBDS, we do not feel that this study had sufficient data to suggest not offering any such imaging as part of planned follow up.

## Conclusions

This study adds to the published literature describing the natural history of small CBDS identified on IOUS in patients undergoing cholecystectomy. Such patients may be safely treated with short-term expectant management including postoperative biliary imaging (6-week MRCP). This is associated with a reduced hospital total length of stay compared with patients undergoing immediate/planned bile duct clearance. The current study provides rationale for undertaking further prospective studies to establish short-term expectant management as a preferred management strategy with the potential for reduced procedure-related morbidity, LOS and hospital costs.
